# Tools for knowledge acquisition within the NeuroScholar system and their application to anatomical tract-tracing data

**DOI:** 10.1186/1747-5333-1-10

**Published:** 2006-08-08

**Authors:** Gully APC Burns, Wei-Cheng Cheng

**Affiliations:** 1 Information Sciences Institute, 4676 Admiralty Way, Marina Del Rey, CA 90292, USA; 2 Neuroscience Research Institute, Univeristy of Southern California, 3641 Watt Way, Los Angeles CA 90090-2520, USA

## Abstract

**Background:**

Knowledge bases that summarize the published literature provide useful online references for specific areas of systems-level biology that are not otherwise supported by large-scale databases. In the field of neuroanatomy, groups of small focused teams have constructed medium size knowledge bases to summarize the literature describing tract-tracing experiments in several species. Despite years of collation and curation, these databases only provide partial coverage of the available published literature. Given that the scientists reading these papers must all generate the interpretations that would normally be entered into such a system, we attempt here to provide general-purpose annotation tools to make it easy for members of the community to contribute to the task of data collation.

**Results:**

In this paper, we describe an open-source, freely available knowledge management system called 'NeuroScholar' that allows straightforward structured markup of the PDF files according to a well-designed schema to capture the essential details of this class of experiment. Although, the example worked through in this paper is quite specific to neuroanatomical connectivity, the design is freely extensible and could conceivably be used to construct local knowledge bases for other experiment types. Knowledge representations of the experiment are also directly linked to the contributing textual fragments from the original research article. Through the use of this system, not only could members of the community contribute to the collation task, but input data can be gathered for automated approaches to permit knowledge acquisition through the use of Natural Language Processing (NLP).

**Conclusion:**

We present a functional, working tool to permit users to populate knowledge bases for neuroanatomical connectivity data from the literature through the use of structured questionnaires. This system is open-source, fully functional and available for download from [1].

## Background

Although massive bioinformatics databases support the activities of researchers by providing immediate access to genetic, molecular and pathway data, most biomedical knowledge is available to researchers only in the form of research articles in the primary literature. The usage of this information is therefore greatly hindered by the process of having to locate, read, understand and synthesize these data. In general, more time is spent by biomedical researchers reading, reviewing and writing scientific reports than on direct experimental effort [2,3]. This situation applies to all disciplines of biology, and suggests that informatics solutions that assist biomedical scientists interact with and synthesize information from the primary literature could potentially have a large impact on the field as a whole.

In specific domains, such as connectional neuroanatomy, the data described within the literature are relatively homogeneous and easy to model within a static database schema [4-6]. In order to perform meta-analysis of the state of knowledge in the field, researchers constructed repositories of domain-specific data entered by hand from the literature [5-14]. These neuroanatomical data were treated as a mathematical graph which could then be analyzed using multivariate statistical methods [10,12,13,15-21]. Analyses of the connections of cat cortex predicted the existence of plaid-pattern sensitive cells in the anterior ectosylvian sulcus demonstrating the potential utility of this approach [10,22].

In spite of apparent 'success stories' such as these, and the obvious importance of providing large-scale Knowledge Bases (KBs) to the community for such generally-useful data as neural connections, the development and population of these systems has always been problematic. In general, Knowledge Representation (KR) solutions should provide specific key functions including intelligent reasoning, efficient computation and human communication [23]. By far, the most significant challenge facing KB builders is to provide efficient and accurate methods of populating the knowledge repository from the literature using so-called 'Knowledge Acquisition' (KA) methods. At the time of writing, there are two large-scale collations of neural connectivity data available to the community via the internet: the CocoMac system [5] and 'BAMS' [6]. To indicate the difficulty of this problem, the process of collating information into CoCoMac is documented in an 89-page booklet (available from [24]). Both systems contain tens of thousands of individual connection reports taken from hundreds of papers (which, in both cases, is still only a relatively small sample of the available literature). Both systems are specialized relational databases and all curation tasks are performed manually by the members of each project. This involves reading papers, interpreting their contents and then entering data into the system to represent the collator's interpretations (with additional annotations to explain the underlying reasoning). This process is essentially a 'data-entry' task that requires the participation of expert curators. The inefficiency of literature-driven knowledge acquisition is evident by comparing this situation with that found in molecular biology where high-throughput acquisition methods provide direct access to data. UniProt [25] is an example that contains billions of individual records. Clearly, the curation of biomedical knowledge from the literature by small specialized teams is insufficient to solve the problem of constructing comprehensive computational representations of the information contained in the literature.

It is important to note that this situation is repeated within almost every biological discipline where a large number of individual facts are reported in the literature. Within this project, connectional neuroanatomy acts only as an *illustrative case *simply because there have been repeated attempts to construct databases in this domain, and none of the existing systems have solved the data entry issue. This paper specifically targets the issues involved with collating tract-tracing experiments, but also provides a general solution that is also concerned with broader issues of how one would construct knowledge bases for other domains.

In this paper, we address this issue within a software application called 'NeuroScholar'. This is a biomedical informatics knowledge management system for scientists dealing with the published literature [14] and their own data [26]. It has been specifically designed for systems-level neuroscience work, but could conceivably be used in any biomedical subject. It is an open-source Java application, available from [1]. The system's design is based on principles of knowledge engineering that incorporate industry-standard object-oriented concepts (based on the Unified Modeling Language or 'UML') and can be translated to frame-logic or predicate-logic KR standards (such as the Web Ontology Language, 'OWL'). The target audience of this paper is the community of biomedical informatics researchers who view the published literature as a valuable resource and who wish to construct KBs of published information. The NeuroScholar system could act as a platform for the development of such systems.

This project is motivated by the observation that the task of carefully reading and annotating research articles is performed many times by scientists in the everyday execution of their work. In fact, researchers individually synthesize a large amount of information into their own personal knowledge representation. This may be entirely based on a capability to understand, memorize and recall the information. Alternatively, this may involve paper notebooks, file-cards or other non-computational strategies. Commonly, scientists will use either home-made or commercial computational knowledge management strategies such as spreadsheets, the construction of summary diagrams in drawing applications (*e.g*., PowerPoint, Adobe Illustrator, *etc*)., databases, Laboratory Information Management Systems ('LIMS'), or note-taking programs (*e.g*., Lotus Notes, OneNote, *etc*.).

Here, we provide a 'study-tool' for biomedical scientists to manage their interpretations and observations derived from the primary research literature as both unstructured and structured annotations. Our ultimate objective is to provide a user interface that supports users' study of the literature and in so doing, provides a medium for easy knowledge acquisition into either personal or communal KBs. Here, we describe the NeuroScholar system as a piece of software supporting the action of constructing individualized KBs for biomedical scientists. This includes the following components: (a) knowledge representation design tools; (b) literature management capabilities, (c) three different methods of literature annotation (free-text, attribute-value pairs and structured KR data); (d) aggregating structured annotations based on multiple text fragments into KRs of complete experiments. We will discuss possible methods of knowledge sharing and consolidation built onto this software. We also suggest that this work is the first step of a strategy that will culminate in the use of text mining to automate knowledge acquisition from published literature.

The work described here has been reported in preliminary form as conference posters [27,28].

## Implementation

### System architecture

**Figure 1:**
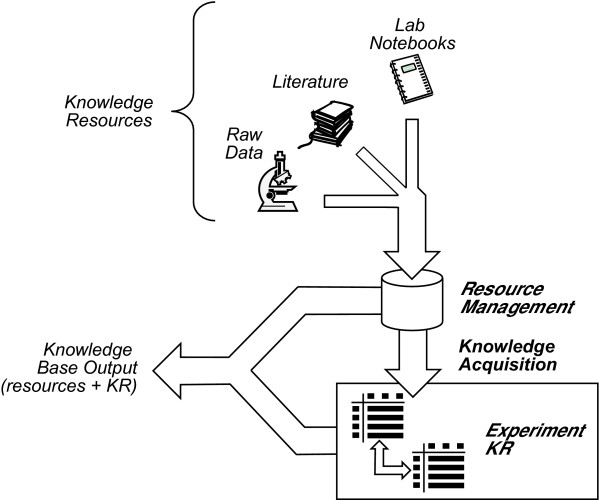
Data flow diagram illustrating the different stages of representation currently implemented within the latest release of the NeuroScholar system.

Data flow within the NeuroScholar system is organized within the architecture shown in Figure 1. At this stage of the system's development, we focus on the task of generating KRs from free-formatted information describing primary data (experimental papers, scanned notebook pages and data images). The system has three components: a repository for the knowledge resources themselves, simple KA mechanisms based on annotations, and a KR component to provide a structured representation of the primary findings of the data.

The repository provides an interface with the PubMed interface of the National Center for Biomedical Information (NCBI) as a simple method to retrieve citations of full-text papers. An incidental observation that has proven to be very useful within the design of NeuroScholar, is that it is only necessary to know four pieces of information (the surname of the first author, the year of publication, the volume number, and the first page of the article) to retrieve a unique PubMed entry for any given citation paper reliably. This structure provides a structure of indexing research articles and provides us with a rapid, easy method of retrieving full PubMed citations with a minimal set of search criteria.

The Knowledge Acquisition component is based on 'Fragmenting' papers (see [26] for a discussion of this process). This is essentially the same process as highlighting an important section of text in an article with a marker, thus delineating that passage of importance to the user. The system then permits the user to attach structured data to these fragments. According to the principle that an efficient KA system must act as a 'good student' by actively trying to acquire knowledge, we use questionnaires to direct the user to enter data concerned with specific issues [29]. The resulting system is an interface that may be used by domain experts with minimal training to populate a KB with structured information.

By retaining the coordinates of the fragment within the context of the original paper, in most cases, it is possible to extract the text being annotated. In cases where this is impossible, (such as with scanned documents where each page is simply a bitmap image) it is straightforward to capture and save an image of the delineation within the PDF file. Optical Character Recognition (OCR) software may then be applied to this image to attempt to reconstruct the text of the fragment. If this may not be permitted (for example, because of copyright restrictions), then at the very least, we may reconstruct the fragment by redrawing the delineations over images of the pages of the PDF file itself. Thus, as long as the end-user has the rights to the original article, any fragments saved in NeuroScholar may be retrieved. In the authors' experience of developing systems that summarize information from the literature, it is essential to retain the *original text *from the source that supports the basis for any subsequent summary [11]. This 'paper-trail' is vital to permit validation and verification of knowledge within the system.

The KR generated uses the 'View-Primitive-Data-Model framework' ('VPDMf', [14,30]) to define composite objects from an object-oriented method of combining packages, classes, associations, roles and attributes [31] into encapsulated data structures called 'views'. The system also permits other object-oriented concepts such as inheritance and dependency to be incorporated into a knowledge model. NeuroScholar provides a graph-like user interface for displaying knowledge representations where views are defined as nodes in a graph and links or relations between views appear as linking edges.

The VPDMf is an automated scripting process that takes a UML model as input with additional XML-based design documents that describe the detailed composition of entities making up the knowledge model. It transforms the UML-based object-oriented design into a relational database schema with a corresponding Java object model based on the same design. This provides NeuroScholar with programmatic access to the data in the schema. We have found that the system can accommodate individual views with quite a complex structure, for example: the tract-tracing-experiment view involves 12 separate classes in the UML model, and a typical view instance contains over 100 individual data tuples in the system's underlying database. The VPDMf has been described as a refereed conference paper in [30]. We use a modular design, so that all components of the latest version of the KB (consisting of 38 data- and 6 relation-views) are organized into six smaller modules (based on 'packages' in the underlying UML model). Each of these governs a different component of the overall system: bibliographic resources and fragments; notebooks and notebook fragments; neuroanatomical components; and representations of specified experiment types.

### Knowledge representation design

**Figure 2:**
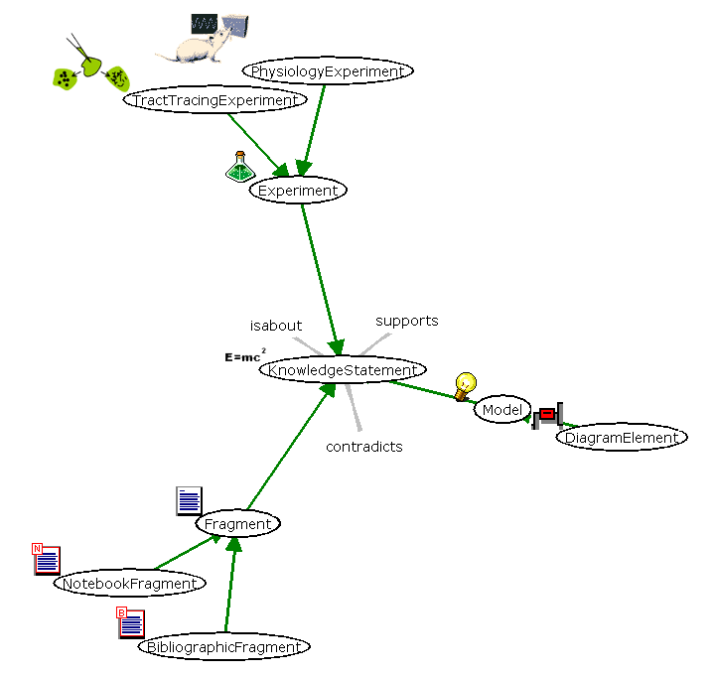
The view definition graph (captured as a screenshot) of the view neighborhood surrounding the 'knowledge-statement' view. This is the central KR construct of the system. Note that the view shown is a slightly magnified view of the graph definition panel in NeuroScholar's user interface.

The central structure of NeuroScholar's knowledge representation is shown in Figure 2 as a screenshot from the system. The system uses the TouchGraph open source library [32] to provide a dynamic, interactive view for the knowledge representation. The 'knowledge-statement' view provides a general representation of a fact or an interpretation. We define three specializations of knowledge-statements: 'fragments' (statements derived from sources external to NeuroScholar), 'experiments' (local representations of primary experimental data) and 'models' (interpretative statements about the meaning of experiments). Both fragment and experiment views are then specialized in turn to provide 'bibliographic-fragments' (fragments derived from literary sources); 'notebook-fragments' (fragments derived from scanned notebooks or data images); 'tract-tracing-experiments' (representations of experiments concerned with studying neural connections) and 'physiology-experiments' (representations of experiments concerned with correlating neural activity with behavior under specific experimental manipulations). As shown in Figure 2, different types of knowledge-statement are the central currency of the system. These views may be linked together by 'supports', 'contradicts' or 'is-about' relations based on ontological designs for argumentation networks [33,34]. For descriptions of the design principles and data models underlying this representation, see [4,35]. As this project is subject to change as solutions and systems are further developed, we refer the reader to the project website for updated schemata and specifications [1].

**Figure 3:**
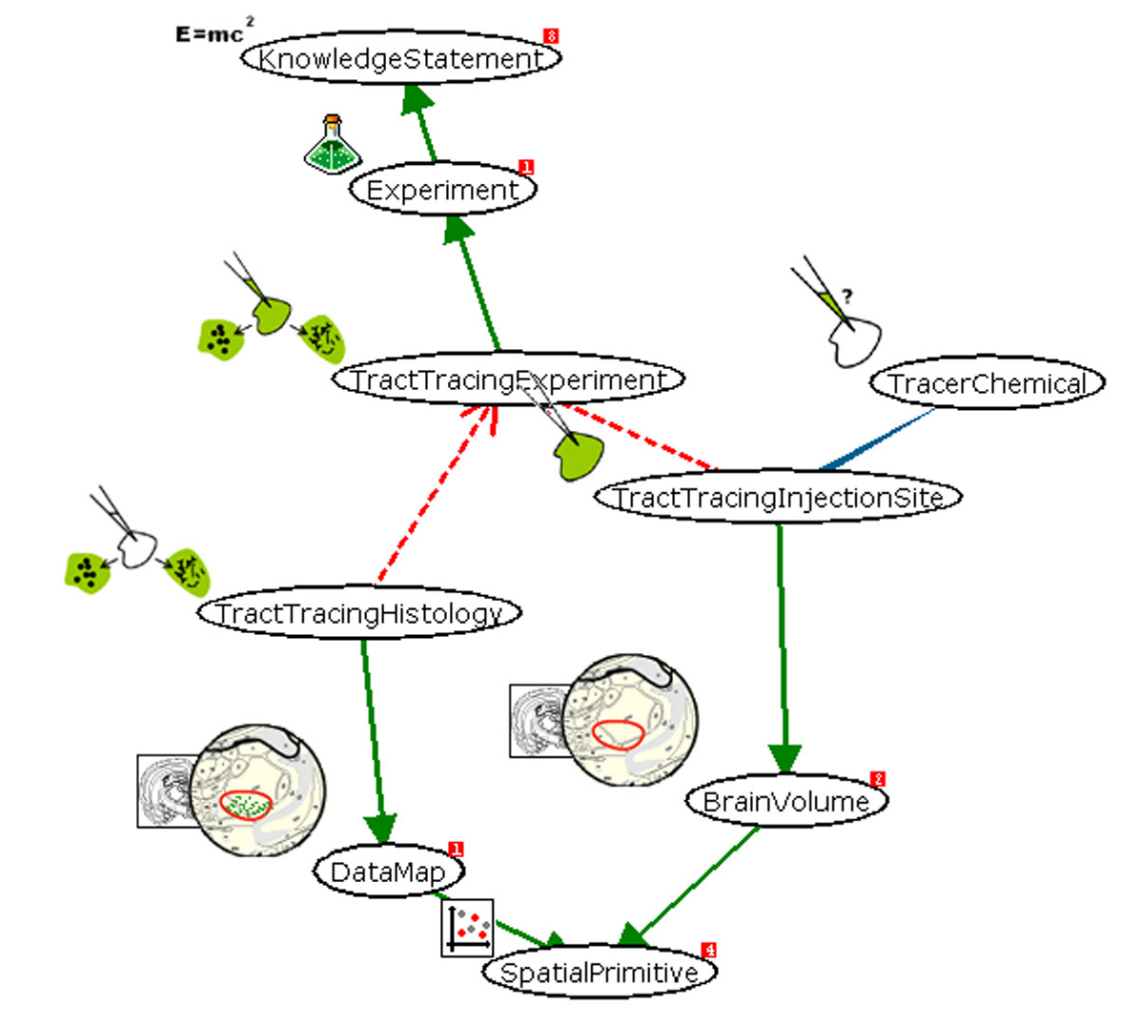
The view definition graph (captured as a screenshot) of the 'tract-tracing-experiment' view. This figure shows the relations between the views supporting the instantiation of the experiment view. Note the use of UML notation (inheritance and dependency arrows).

Within this paper, we report data concerning the use of the system to collate information from the tract-tracing literature. See [35,4] for the conceptual and logical design of the underlying KR. The View-Definition-Graph of this representation is shown in Figure 3.

There are three views which have significance for this representation (A) the 'tract-tracing-experiment' (TTE) view, (B) the 'tract-tracing-histology' (TTH) view and (C) the 'tract-tracing-injection-site' (TTIJ) view. The TTE is a child of the 'experiment' and 'knowledge-statement' views (which permits it to be incorporated into argumentation networks through the use of 'supports' and 'contradicts' relations, see Fig 2). The TTH view reflects a pattern of histological labeling across the brain (as shown in neuroanatomical maps that may be added to the system via the NeuARt II plugin [36]) so that it inherits from the 'data-map' and 'spatial-primitive' views. Conversely, the TTIJ view reflects a small region of tissue where the initial deposit of tracer is made (and may also be described using the NeuARt II plugin, see later); therefore, it inherits from the 'brain-volume' and 'spatial-primitive' views. The TTIJ view is also linked to a 'tracer-chemical' view, which represents a 'model' view, this denotes the type of tracer used in the experiment (*e.g*., Phaselous leuco-agglutinin or 'PHAL'; Fluoro-Gold or 'FG'). Both the TTH and TTIJ views are dependent on the TTE view so they cannot exist in isolation from the experiment itself. In addition, the TTH and TTIJ views are actually included in the TTE view itself, (illustrating the capability of the VPDMf system to be able to capture the design of composite knowledge structures as a single encapsulated object).

These entities comprise the minimum information that is necessary to be able to infer the essential qualities of a neural projection from this type of experiment. An injection is made in a specific region, and produces transported labeling in a number of other regions. The uptake properties of the tracer chemical used in the experiment determine the direction of axonal transport used by the labeled neurons ('anterograde' or 'retrograde') and therefore reveal the direction of the neural connection. This structure is consistent over experiments that use different, non-transynaptic tracers.

**Figure 4:**
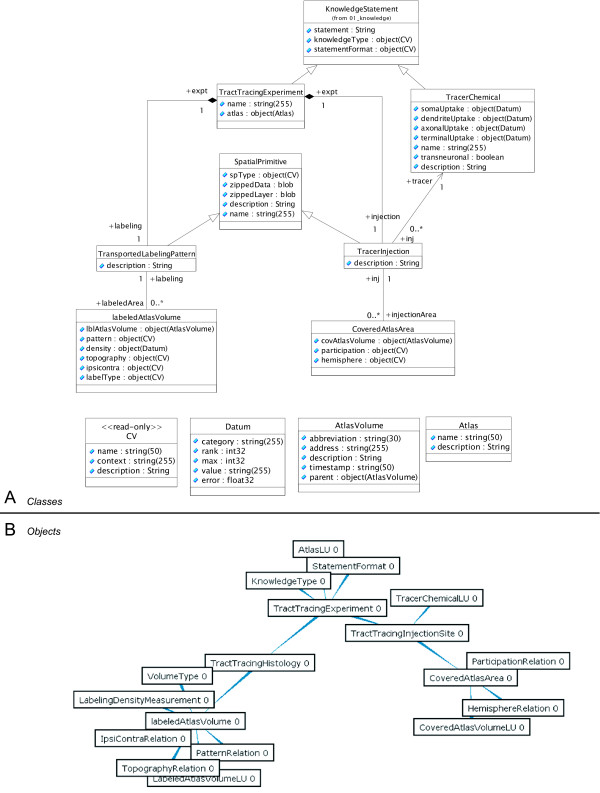
Internal class- (and object-) structure of the tract-tracing experiment view. A: UML class diagram showing all views involved in the definition of the 'tract-tracing-experiment', 'tract-tracing-histology' and 'tract-tracing-injection' views. Attribute notation based on Object Management Group (OMG) standards and is an internal representation of the VPDMf system. B: Screenshot showing the primitive-instance graph of an non-instantiated tract-tracing-experiment. This shows the potential complexity of each transaction with the underlying relational database system.

One of the strengths of the VPDMf as a representational methodology is that complex structured data may be encapsulated into a single view that can be managed and manipulated by end-users. The internal intricacies of an individual view can be hidden from end-users, so that numerous internal components of a complex view (such as TTEs), can be presented in a single form or web-page. We show the TTE view as UML class and object diagrams in Figure 4.

The class diagram shows the current design for the TTE. The abbreviations used for attributes follow the Object Management Group (OMG) types (*e.g*., 'int32' stands for 32-bit-integer, *etc*.) and are mostly self-explanatory. In situations where references to other classes appear as 'object(target)', we have included all target classes in Fig. 4. The 'CV' class stands for 'controlled vocabulary' and is a read-only lookup table for specific terms. The 'Datum' class refers to a specific measurement or value and can be used to represent ordinal data (simply by providing ranks). Two additional classes are included for atlas-based spatial data: the 'atlas' class itself (*e.g*., 'Swanson92' is the identifying code for reference [37]) and the 'AtlasVolume' class to refer to each named structure in a given atlas. Within the VPMDf, the full structure of an instantiated view is represented as a graph with interconnected nodes. The object diagram shown in Fig. 4 is derived from a screenshot of the functional system. It illustrates all the objects involved in an 'empty' TTE view and provides a concrete representation of the object-to-object connectivity. Typically, a TTH view captured by the system from a research article could involve as more than one hundred separate 'labeledAtlasVolume' primitives (see Fig. 4), so that with a real example, this graph would be unreadable.

### General user interface design

**Figure 5:**
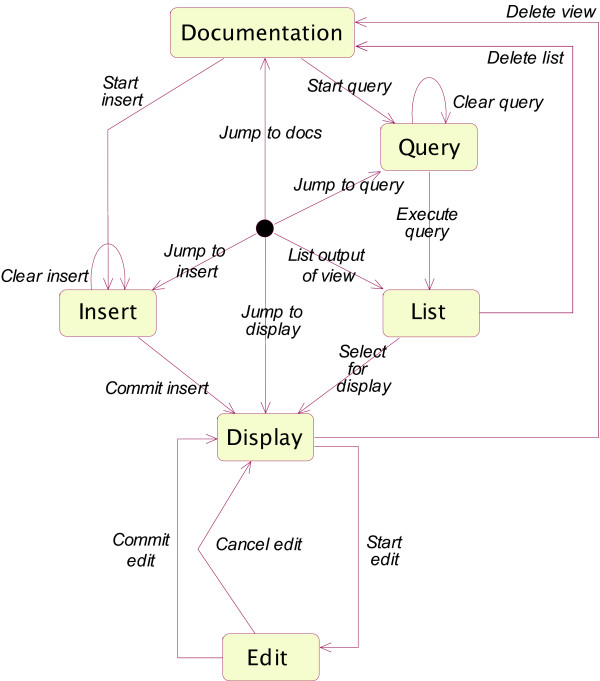
UML State Diagram showing the main states and transitions within the NeuroScholar system. Each state-change corresponds to an action performed as a single transaction on a view (such as that shown in Fig. 4).

Since web-surfing is universal and intuitive for almost all computer users, we based the 'look-and-feel' of the NeuroScholar interface on that of a web browser. On system startup, users are presented with a clickable list of both active and archived KBs. Upon selecting an active knowledge base, users are then presented with a list of views within the system (with counts of instances of each view). All subsequent actions are governed by the state machine shown in Fig. 5 (as a UML state diagram where polygons are system states and arrows are actions that cause transitions between states).

The user interface is always in one of these states for a specific view, except at system startup, when the user has not yet selected a current knowledge base. Actions may be triggered by menus, popup menus, clickable links, or buttons. The TouchGraph interface shown in Fig. 2 provides another medium for interaction by right-clicking (or shift-clicking) nodes and edges for view-specific interactions. In order to alter the contents of the knowledge base (by entering the 'Edit' or 'Insert' states, users must first log in to the system. This state-based system allows us to implement a history function so that users may view actions they have taken within the system and return to previous states; this deliberately mirrors a web-browser's 'back-button' and 'history' features.

### Programming specifics

The system is developed in Java 1.4.2 and may be run on Windows, Mac OS X or Linux. It uses the MySQL open source relational database as its persistence mechanism. We have implemented a web-services tier for network access to the underlying knowledge base. The complete source code of the NeuroScholar system is available from the project page on SourceForge [38] and the program is deployed as an installer package, (see [1] for documentation about downloading and installing the system). This process may require expertise in setting up the MySQL database with an administrative password (we describe this process and other common set-up tasks with movie documentation found on the website). The system is supported by an extensive testing framework based on JUnit [39] which iterates through every state and action shown in Fig. 5 (insert, delete, edit, query, list, *etc*.) for every view in a specific model and reports exceptions and failures. We have tested the core elements of the system for each platform described above, through the use of this framework. The Fragmenter module uses the excellent Multivalent Java library to render and parse PDF files [40,41].

## Results

**Figure 6:**
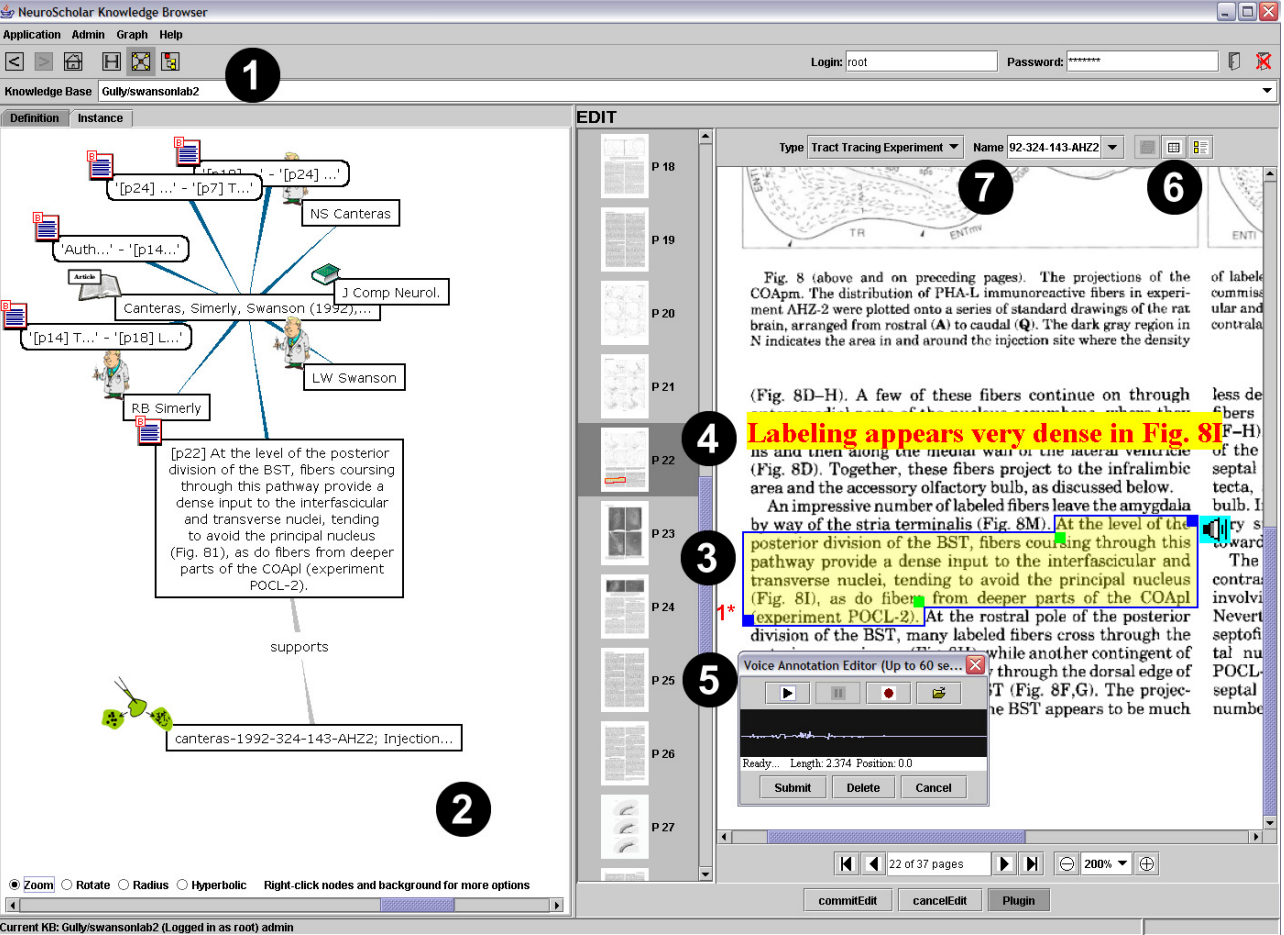
Labeled screenshot of the functional NeuroScholar system. Labeled numbers within the figure correspond to the following subsystems (1) toolbar and menus; (2) graph panel; (3) a fragment; (4) a text annotation; (5) a voice annotation; (6) attribute/value pair-based annotations; (7) the 'knowledge capture' subsystem. See main text for full descriptions of these subsystems.

The NeuroScholar system provides several sets of features that enable users to interact with full-text scientific articles, all centered on the 'Fragmenter' plugin. These interactions are based on (a) simple textual and voice-based annotation functions; (b) attribute-value pair annotation functions; and (c) the 'knowledge capture' subsystem (which forms the principle contribution of this paper). We will describe each of these systems in turn. The appearance of the NeuroScholar system displaying a 'bibliographic-fragment' view with the fragmenter plugin active appears in Fig. 6. In order to illustrate a number of functions being invoked in this image, we have placed numbered labels in the figure to identify different sections.

### (1) The toolbar and menus

The system has a similar overall layout to common web-browsers, so that the 'address bar' contains the name of the current knowledge base, there is a button for the homepage and buttons to move backward and forward through the history of views displayed in the application (rather than a history of visited web-pages) and there are a set of three toggle buttons that open a panel on the left-hand-side of the main view to provide additional functionality: the History panel, the Graph panel or the and Tree panel.

### (2) The graph panel

As described above, pressing one of the three toggle buttons in the toolbar activates the left-hand-side panel with one of three user interfaces. The History panel permits the user to return to a previous view/state combination (such as 'displaying the bibliographic-fragment with the Unique ID value of 33146', as is the case for the view shown in Fig. 6). Fig. 6 shows the Graph panel, which contains two tabs. One is used to display the view definition graph (shown as isolated images in Figs. 2 and 3 to illustrate the design of the knowledge representation) while the other displays the view instance graph (shown in Fig. 6). Consistent with graphical conventions from the semantic web, classes are shown as ellipses and instances as rectangles [42]. The example shown is taken from [43], showing only 1 of 97 fragments derived from this paper. NeuroScholar attempts to alleviate the visual clutter of having a large number of nodes presented in the graph at one time by providing 'proxy-nodes', which are lozenge-shaped and 'contain' 30 alphabetically-sorted view instance nodes. Each view instance node in the graph shows only the first line of its index, unless the user places the mouse over the node to reveal the full text of the node's index (see the central node in Fig. 6). Right-clicking a view instance node provides more functionality: the user can list all views of a specific type that are linked to the node. Double clicking a node will display the view instance in the main panel. In some situations, the graph panel can become cluttered with a large number of views. The Tree panel offers an alternative way of browsing views based on the standard design of a tree-based interface component (similar to Windows Explorer on a PC, not illustrated).

### (3) The fragment

The image shown illustrates the structure of the delineation of an excerpt (as part of a 'bibliographic-fragment' view in NeuroScholar). The polygon used to create the fragment is made up of four points: users select the enclosing rectangle of the text of interest by moving the top-right and bottom-left blue points, and then they may indent the top-left and bottom-right green points to precisely delineate the text or figures of interest in the article (see Fig. 6). This simple design provides an intuitive interface for this task. If available, the system will use Multivalent's text-extraction capabilities to parse the raw text of the fragment from the PDF file in conjunction with a simple spatial indexing library (in Fig. 6, compare the text of the central node of the view instance graph to that of the excerpt). Each excerpt is numbered since multiple excerpts may contribute to an individual fragment (as is necessary when a sentence or passage of interest extends to multiple pages). The 'asterix notation' denotes that the excerpt contains captured data (see later).

### (4) The text annotation

The simplest form of annotation available to users is to enter their own free-form comments. These can be shown as text superimposed over the article and may be placed above, to the side of, below or inside the delineation. These notes may be searched from within NeuroScholar in the standard way.

### (5) The voice annotation

Another unstructured form of annotation is the ability for users to record one minute of sound and link it to the fragment (see Fig 6).

### (6) Attribute/value pair-based annotations

**Figure 7:**
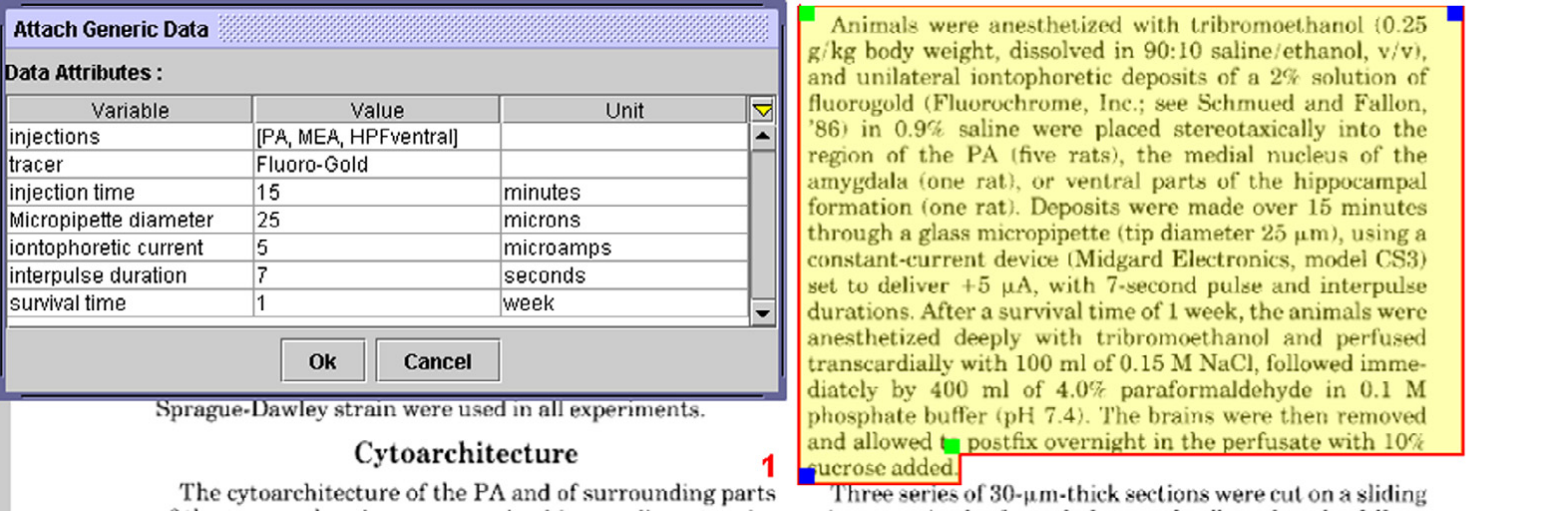
Screenshot showing generic data annotations based on attribute-value pairs. These permit users to structure annotations themselves.

The button labeled in Fig. 6 generates the dialog box shown in Fig. 7, which may be populated with free-form attribute-value pairs (with appropriate units).

Fig. 7 shows some parameters of potential interest to researchers performing tract-tracing work including detailed quantitative parameters. As such, the advantage offered by the system over routinely surveying the literature is that the data are linked directly to the text of the paper and the data variables and values are stored in a database from which they may be queried and retrieved. These data-values are actually part of the definition of the 'knowledge-statement' view and may be searched for within the main NeuroScholar system.

### (7) The 'knowledge capture' subsystem

The main contribution of this paper is the methodology constructed to assist with knowledge acquisition for neuroanatomical tract-tracing data. The objective is to accelerate the speed and ease of acquiring information concerning the minimal information required for a tract-tracing experiment to be interpretable in terms of identifying the origin and termination regions of neural connections and an ordinal account of the connection's strength. At this stage, we are not attempting to capture all details of the experimental method, or the nuanced details concerning the data's reliability.

Knowledge acquisition systems should behave like good students, prompting clarification from domain experts but are not necessarily skilled at structuring the information appropriately in the correct KR [29]. Initially, the user must select the 'type of experiment' from a list and then provide the experimental data set a unique name (which is subsequently used to collate captured information from different fragments). Having identified the experimental type, the sub-system guides the user through the process of data entry by presenting a set of questions, that each correspond to a specific aspect of the complete KR (with a data-entry form). Each question (and corresponding data-entry form) is designed to be answerable from a typical single fragment found in the paper, *i.e*., the question 'describe the injection site' has three components, the name of the regions injected, the extent to which the injection covered those areas, and the side the injection was made. Typically, a description of an injection site in a paper would include this information.

For tract-tracing experiments, we pose four questions:

1. What atlas are you using?

2. Please describe the injection site.

3. Please describe the labeling pattern.

4. What tracer chemical are you using?

**Figure 8:**
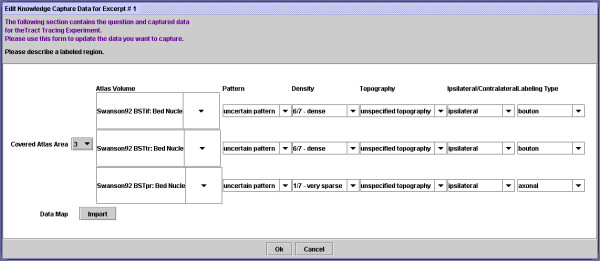
Screenshot showing structured questionnaire form for knowledge capture pertaining to 'tract-tracing-histology' component of a 'tract-tracing-experiment'.

The data entry form for the description of the labeling pattern (question 3) is the most complex and is shown in Fig. 8.

This illustrates how the fragment shown in Fig. 6 is collated into the system. The fragment describes very sparse labeling from a Fluro-Gold injection in the cells of various regions of the Bed Nucleus of the Stria Terminalis. Most of the data entry fields simply contain default values. Note that this data entry form also includes a button to trigger the NeuARt II plugin. This tool is described in detail elsewhere [36] and permits the user to load a detailed three-dimensional map of the labeling pattern into the system if available. This function does not contribute directly to the KR generated within the NeuroScholar system and so will not be described further here.

**Figure 9:**
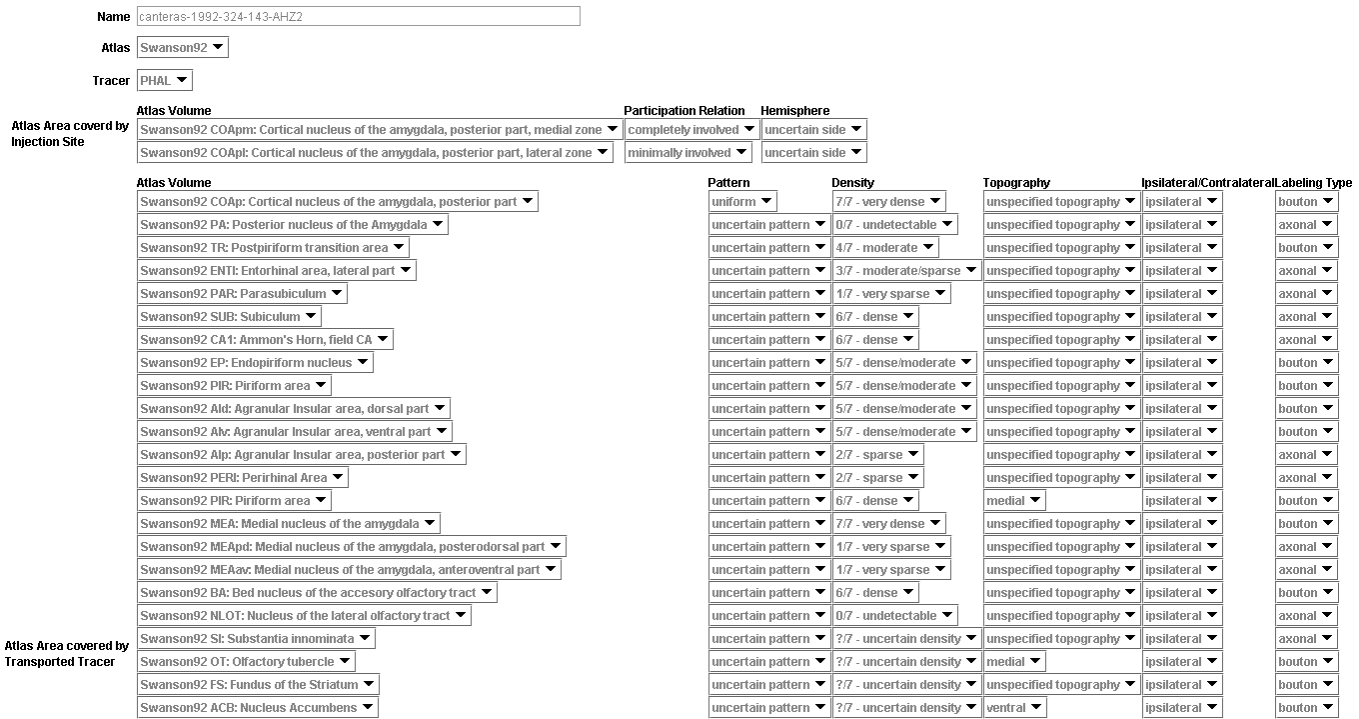
Screenshot showing a partial view of the 'tract-tracing-experiment' captured from experiment AHZ-2 of reference [43].

Finally, upon completion of this knowledge capture process from fragments, the user may insert a new 'tract-tracing-experiment' into the system. This is accomplished by entering the 'insert' state for the 'tract-tracing-experiment' view (see Fig. 5). Normally, the user is presented an empty form to be filled in, but with views supported by the knowledge capture system, the user is asked to select a name from a pull-down list of captured experiments. When the user selects one, the system surveys the whole system for any appropriate captured data that are associated with the selected experiment name. The system compiles these data into a single view instance and then inserts that into the system. Additionally, the system inserts '*supports' *relations between this view and all the supporting fragments that contain the relevant capture data (see Fig. 6). To ensure concurrency, this whole process must be repeated whenever any of the fragments that contribute to the experiment are edited or deleted. The final structure of the form for our example in this paper is shown in Fig. 9. This design captures the most important features of the data for each aspect of the view as well as details that are not always presented in the textual descriptions found in the literature (such as the topography of labeling) but are nonetheless important to include in the final KR.

## Discussion

A key step for building knowledge bases for systems-level biomedical data is acquiring information from the literature. At present there are three approaches that are viable solutions for this problem: (1) curation by a small specialist team; (2) community-based curation; or (3) Information Extraction using Natural Language Processing (NLP). The trade-off of these approaches lies between accuracy, reliability and scalability; ultimately we seek an approach that provides an accurate, complete and relevant set of searchable facts for any given domain of science. A compromise that omits one of these three factors could seriously compromise the usefulness and scientific impact of the system. Within this paper, we describe software that provides support for all three approaches mentioned above. The knowledge capture component directly supports both curation by specialists and the community (since the tool is freely-available and downloadable). Importantly, the approach links the captured data to the text that defines them and the context of that text within a journal article. This is ideal 'training data' for information extraction using statistical NLP, since they provide correct examples of the data associated with specific fragments (see [44] for a recent review of text-mining in Biology and Biomedicine).

Ultimately, knowledge acquisition in biomedicine should contribute to large-scale, global solutions that may be used by the whole community. There are several strategies that could bring this about. (A) Within the human genome project, data acquisition is linked directly to the process of publication. In order to publish papers, authors are *required *to upload the relevant genetic data to shared databases. Publishers could conceivably require that standardized data be uploaded to centralized servers wherever possible as a condition for publication; (B) Weblogs ('blogs') and shared community-based online communities ('wikis') provide a medium for users to post their interpretations online. It is possible that scientists could use this methodology to post comments and interpretations of published work. (C) Central databases for knowledge of a specific type (such as tract-tracing data), which users could upload their information to. (D) Finally, the strategy we suggest is that individual users perform knowledge acquisition tasks on a small-scale as part of their everyday study of the literature.

All large-scale centralized approaches to this field are confounded by the complexity and heterogeneity of the requirements of users and of the representations chosen to serve them. The process of devising a 'standard' representation for any individual domain of biology is a large-scale undertaking. The concept of 'minimum required information' is used by the Microarray Gene Expression Data society (MGED, [45,46]) in their computational representations for microarray ('MIAME') and *in-situ *hybridization ('MISFISHIE') data. These are formal representations that MGED is promoting as standard templates to enable and encourage data sharing between systems [47]. The concept of 'minimum information required' depends strongly on the task that the information would be used for. The process of defining standard representations for classes of experimental results should also include an account of the purpose of the representation. The schema we describe could be considered the minimum information for a tract-tracing experiment to describe the start- and end-points of neural connections correctly. It should be noted however, that this representation does not explicitly describe the details of the experimental method used.

The potential of our approach is that the act of comprehending the data in the literature sufficiently well to be able to place it into a computational representation is repeatedly performed by scientists all the time in the course of their work. If some advantage could be provided to them by entering their understanding of the data into a computational database, the rate-determining step of the curation process would be solved. This paper provides a tool that takes a step towards this goal.

Certainly, this perspective raises important questions: if our approach is based on many experts annotating the text found in the literature, how can we ensure inter-annotator agreement? This is addressed within the field of biomedical Natural Language Processing since most machine-learning approaches use manually-annotated text as their gold standard (see [48]). Improving agreement in NLP research is based on providing carefully written guidelines for annotators to follow. Outside of this structured approach, if the use of the system becomes widespread with a large number of different annotators working on the same text, we would have access to statistical data that could address this question.

Tool development drives small-scale, community-based solutions and the development of the Protégé ontology construction tool [49,50] over the course of the last nineteen years is a success story [51]. Protégé has a very large user group which now contributes to the core technology of the National Center for Biomedical Ontology (NCBO) under Professor Mark Musen. Interestingly, Protégé does not currently implement methods to link the research literature directly to concept definitions and instances. Given that one of the current weakness of NeuroScholar is the lack of integration with standard formats such as OWL, it would be a natural development to use NeuroScholar's knowledge capture mechanism to generate Protégé-based ontologies. Given that both software packages are open-source and implemented in the Java programming language, such a development effort would be relatively straightforward and immediately useful.

Other projects of interest to our current effort include the Neurocommons project [52], which advocates an 'open-content' agenda for neuroscientific knowledge in a community based approach. The Biomedical Informatics Research Network (BIRN) has been very active in developing mediator technology to enable the integration and sharing of research data [53,54], combined with ontology-based approaches to disease maps [55].

Rather than acting as an easily-modifiable KR framework that supports inference and other logic-based functions, this system serves primarily as an instance repository. The encapsulation mechanism used to define views in the VPDMf differs from KRs based in logic since each individual view contains many classes and attributes (including data-oriented structures such as binary objects) for a single entity. This forms the basis of a 'hybrid KR' where concepts that are normally represented in KR systems by a single term (such as a brain region), may be enriched with contextual data (such as the regions' delineation in a brain atlas). KA approaches have been studied in the context of logic-based KR systems, using systems such as LOOM for their internal representations [56]. These systems have become quite sophisticated for tasks such as itinerary planning and may serve as the basis for predicate-logic-based knowledge acquisition in biology [57].

The CommonKADS framework is an example of a knowledge engineering approach that provides a practical set of guidelines as a useful structure for the development of knowledge engineering solutions for specific knowledge-based tasks [58]. Like our system, CommonKADS uses the UML as its foundational language. The purpose of this paper is to contribute to the community both the software as a finished product but also the libraries, source code, specifications and designs that might enable programmers on other projects to perform similar work. We view open-source programming as an essential component of this sort of applied informatics research and exhort our colleagues to not only publish their algorithms but also their source code, documentation, design and data as well.

## Conclusion

Within this system, we provide a knowledge acquisition interface for data curation based on annotation of the primary research literature. We provide one example of the interface's use to curate a knowledge base of data concerning tract-tracing neuroanatomical experiments. We anticipate that this software will enable biomedical scientists to construct small-scale knowledge bases of data relevant to their own personal research questions which could then be accessible by the community as a whole.

## Availability and requirements

Project name: NeuroScholar

Project home page: 

Operating system(s): Windows, Mac OS X, Linux

Programming language: Java

Other requirements: Java 1.4

License: Slightly modified version of GNU GPL (from the University of Southern California)

Any restrictions to use by non-academics: No restrictions

## Competing interests

The author(s) declare that they have no competing interests.

## Authors' contributions

GAPCB & WCC wrote the paper. GAPCB & WCC designed, built and tested the NeuroScholar system. The Fragmenter and Knowledge Capture subsystem were entirely designed and programmed by WCC. The preliminary data presented in this paper was entered into the system by GAPCB.
